# The initial visibility of updated recommendations on preseason heat safety in high school athletics among United States athletic trainers

**DOI:** 10.1371/journal.pone.0300669

**Published:** 2024-03-22

**Authors:** Zachary Yukio Kerr, Jake C. Diana, William M. Adams, Johna K. Register-Mihalik, Aliza K. Nedimyer

**Affiliations:** 1 Department of Exercise and Sport Science, University of North Carolina at Chapel Hill, Chapel Hill, NC, United States of America; 2 Human Movement Science Curriculum, University of North Carolina at Chapel Hill, Chapel Hill, NC, United States of America; 3 Department of Sports Medicine, United States Olympic & Paralympic Committee, Colorado Springs, CO, United States of America; 4 United States Coalition for the Prevention of Illness and Injury in Sport, Colorado Springs, CO, United States of America; 5 Department of Kinesiology, University of North Carolina at Greensboro, Greensboro, NC, United States of America; 6 School of Sport, Exercise and Health Sciences, Loughborough University, Leicestershire, United Kingdom; Portugal Football School, Portuguese Football Federation, PORTUGAL

## Abstract

Updated recommendations on preseason heat safety in high school (HS) athletics (“2021 Consensus Statements”) were published in April 2021. This cross-sectional survey study explored the initial roll-out of the 2021 Consensus Statements, including their visibility among United States HS athletic trainers (ATs) and perceived levels of confidence in implementing them. Recruitment occurred first, from a random selection of ATs from the Board of Certification, Inc., and second, an open invitation via social media. An online cross-sectional questionnaire had participating ATs note whether they had seen the 2021 Consensus Statements. If yes, ATs reported their perceived level of confidence in implementing them (5-point-ordinal scale from “not at all confident” to “very confident); if no, ATs disclosed (open-ended) why they had not yet seen them. Descriptive statistics were calculated for quantitative variables; template analysis identified codes related to visibility of and confidence in implementing 2021 Consensus Statements. Nearly half (45.7%) of 116 responding HS ATs reported having seen at least one 2021 Consensus Statements; 23.3% had reviewed all three. Common reasons among the 63 that had not seen them included: not aware they were published (n = 22), have yet to read them (n = 19), and believed they could not access the journal (n = 10). Of the 53 ATs having seen at least one of the 2021 Consensus Statements, 67.9% (n = 36) were very/fairly confident in implementing them at their HS. Reasons for confidence included their schools ensuring up-to-date EHI prevention and management practices (n = 18) and athletics constituent support (n = 8). This exploratory study observed proportions of surveyed HS ATs that had not seen the 2021 Consensus Statements and were not confident in implementing them. Findings highlight the need to continue improving messaging about access to best-practice recommendations. Further, continued efforts inclusive of active and passive dissemination strategies across all athletics constituents are needed to aid proper implementation.

## Introduction

The Centers for Disease Control and Prevention (CDC) classifies athletes as a group that is disproportionately affected by adverse outcomes associated with extreme heat [1]. Further, changing climates may place more individuals at risk for exertional heat illness (EHI) [2]. This is of particular concern for high school (HS) sports due to its large population of over 7.8 million participants [3]. Although EHI in HS sport settings occur across different sports and geographical settings, the large majority are present in American football [4–7]. especially during preseason practices (July and August).

The National Athletic Trainers’ Association (NATA) created an Inter-Association Task Force (IATF) with the goal of reducing the risk and incidence of EHI in high school sport settings. Through the use of epidemiological data, content-area experts, and biological and clinical evidence from lab and applied scientific research, the NATA-IATF wrote preseason heat acclimatization guidelines which focused on gradual acclimatization to the environment during the first two weeks of the football preseason [8]. While these guidelines were found effective in reducing EHI events when state HS athletic associations mandated their implementation [2], research suggests compliance with this and other exertional heat illness prevention strategies have been limited [9–15].

More than a decade has passed since these preseason guidelines were developed. The need for updated information to address potential gaps, alongside acknowledging implementation-related barriers, led to a series of roundtable discussions with experts in 2019. This resulted in the publication of a series of manuscripts providing updated recommendations on preseason heat safety in HS athletics in the April 2021 issue of the Journal of Athletic Training (JAT). The three “Roundtable on Preseason Heat Safety in Secondary School Athletes” publications focused on: “Environmental Monitoring During Activities in the Heat,”[16] “Heat Acclimatization,”[17] and “Prehospital Care of Patients with Exertional Heat Stroke.”[18] These publications are henceforth known as the “2021 Consensus Statements.”

As evidence-based best-practice documents are created and refined, it is essential to continue evaluation and data collection efforts to identify how to best implement prevention strategies. Frameworks exist to help contextualize this evaluation process [19], with injury-specific frameworks including the sequence of prevention [20] and the Translating Research into Injury Prevention Practice (TRIPP).[21] Although each of these frameworks includes distinct qualities, they all advocate for the continual examination of the adverse outcome of interest and the contexts in which the preventive strategy is effective. Further, they emphasize the longitudinal process of evaluation. Thus, it is important to investigate if and how the 2021 Consensus Statements have been seen and interpreted by ATs in its initial roll-out. The current study is exploratory in nature and aims to answer the following research questions:

- RQ1: What proportion of United States (US) HS ATs have seen the 2021 Consensus Statements?
- RQ2: Among US HS ATs who had seen the 2021 Consensus Statements, what was their confidence in implementing them?
- RQ3: What factors among US HS ATs were associated with having seen the 2021 Consensus Statements?
- RQ4: What factors among US HS ATs were associated with having felt fairly or very confident in implementing the 2021 Consensus Statements?

## Materials and methods

This cross-sectional study was part of a larger multi-method parent study focused on information seeking and implementation practices related to EHI among HS and collegiate Ats [22].

For this particular study, the population of interest was ATs working in US HS settings. The measurement items for this specific study were only seen by those denoting they were HS ATs. Further, these items were included in order to conveniently obtain data from a sample of HS ATs, all of whom were recently introduced to the 2021 Consensus Statements.

Data were collected during November 10, 2021-January 31, 2022. The study was approved by the Institutional Review Board at the University of North Carolina at Chapel Hill. All participants provided written informed consent.

### Sample and recruitment

Inclusion criteria for the study consisted of: 1) practicing ATs within the US; 2) certified by the Board of Certification, Inc. (BOC); 3) employed within HS settings. Recruitment occurred in two phases. In the first phase, 4524 HS ATs were randomly selected from the BOC listserv of ATs that satisfied the inclusion criteria. These ATs received an email invitation to participate in the study. The email was sent in two waves with one-half of the ATs receiving the initial email invitation and the other half receiving the initial email invitation three weeks later. The decision to send invitations in two waves was made *a priori* to minimize potential burden to the study team as part of the larger study [22]. A follow-up reminder email was sent three weeks after the initial email invitation was sent. The first recruitment phase resulted in data from 113 ATs (2.5% completion rate). The second recruitment phase involved the primary investigator (AKN) posting the invitation to participate on social media. The post was re-posted a week later, with the survey closing the following week. The second wave resulted in data from 3 ATs. A check was conducted based on data provided by participants and geolocation information provided by the questionnaire host, Qualtrics (Qualtrics Labs; Provo, UT) to determine whether the 3 data points from social media recruitment could be duplicate responses from the BOC listserv. With no such concerns raised, the final overall sample was 116 HS ATs.

### Online questionnaire

The online questionnaire was distributed via Qualtrics. DeVellis guidelines for scale development were used to guide questionnaire development from conception to completion [23]. As part of this, prior to its deployment, the questionnaire went through a content evaluation phase in which it was reviewed by seven content experts with experience in survey development and backgrounds in athletic training, epidemiology, organization behavior, and/or implementation science. The experts provided feedback on the face and content validity, relevance, and clarity of questionnaire items. Adjustments were made before piloting the questionnaire to five practicing ATs (two HS and three collegiate). Feedback was incorporated into the final version of the questionnaire. Measures pertinent to the current study are described below.

At the start of the questionnaire, demographic information and educational background for the ATs were collected. In addition, clinical practice information and current job setting were obtained. Data of interest for the current study included: 1) age in years; 2) gender; 3) race/ethnicity; 4) advanced degree (originating from fields including but not limited to athletic training, exercise science, kinesiology, sport administration, public health, health administration); 5) years of practice as an AT; 6) direct employment by HS/district (versus outside employment such as a hospital/PT company); 7) HS student enrollment size; and 8) zip code of HS.

At the end of the questionnaire, exploratory questions were provided to examine ATs’ knowledge of the 2021 Consensus Statements. First, ATs were provided with links to the 2021 Consensus Statements (within the JAT website) and asked: “Have you seen the updated recommendations on preseason heat safety in secondary school athletics published in the April 2021 issue of the Journal of Athletic Training?” ATs were asked to answer considering if they had seen the 2021 Consensus Statements prior to taking the questionnaire. Those who responded, “Yes” were asked about which of the three specific publications they reviewed, their perceived level of confidence in being able to implement them (5-point-ordinal scale from “not at all confident” to “very confident), and why they chose the specific ordinal scale response (via free-response item). Those who responded “No” were then asked (via free-response item) why they had not yet seen the updated publications.

### Statistical analysis

All analyses were conducted with SAS v9.4 (SAS Institute Inc.; Cary, NC). Descriptive analyses were conducted for all measures of interest.

#### Research question 1

To address RQ1 (What proportion of US HS ATs have seen the 2021 Consensus Statements?), frequencies were computed in regard to the proportion of participants that reported 1) having seen at least one of the 2021 consensus documents, and 2) all three consensus documents. Frequencies were also computed for the proportion seeing each publication singularly. In addition, coding was conducted among those noting they had not seen the 2021 Consensus Statements to further explore responses from the free-response item related to why ATs had not seen the 2021 Consensus Statements (see below).

#### Research question 2

To address RQ2 (Among US HS ATs who had seen the 2021 Consensus Statements, what was their confidence in implementing them?), frequencies were computed in regard to each perceived level of confidence in implementing the recommendations. This analysis was done solely among those participants who had seen the 2021 Consensus Statements. In addition, coding was conducted to further explore responses from the free-response item related to why ATs who noted having seen the 2021 Consensus Statements chose the specific level of confidence in implementing them (see below).

#### Coding of free-response items in RQs 1 and 2

Coding was performed by two members of the research team (ZYK and JCD) with different levels of previous exposure to the study topic. ZYK is an injury epidemiologist that has engaged in work regarding the evaluation and dissemination of sports medicine and injury prevention policy. JCD is a doctoral student with a background in exercise physiology and less exposure to sports medicine and injury prevention policy research.

The open-ended item data were coded with each RQ utilizing its own coding process. To begin, the coders identified *a priori* codes that might be relevant based upon previous literature on initial roll-outs of prevention materials as well as dissemination with sports medicine/injury prevention-related settings [11, 12, 15]. An initial examination of a random subset of responses were then reviewed, with additional codes added to the codebook. This codebook was then used in consideration of the coding of all other responses. However, as data coding ensued, the qualitative concept of “constant comparison” was applied, in which emerging codes were compared with previously coded data in order to continuously create the most relevant codes. All changes made to the codebook were then retroactively applied to all previously coded responses.

Given the exploratory nature of this study, codes were also considered in the context of the previously described descriptive analyses in RQs 1 and 2 to assess their comparability to previous research as well as their plausibility to the findings. Thus, counts for each code were tabulated to indicate how common we identified these appearing within the sample.

#### Research questions 3 and 4

A series of statistical analyses were run to address RQ3 (What factors among US HS ATs were associated with having seen the 2021 Consensus Statements?) and RQ4 (What factors among US HS ATs were associated with having felt fairly or very confident in implementing the 2021 Consensus Statements?). For RQ3, two outcomes were assessed: 1) the proportion of ATs seeing at least one of the 2021 Consensus Statements; 2) the proportion of ATs seeing all three of the 2021 Consensus Statements. For RQ4, the assessed outcome was: 1) among those noting seeing at least one of the 2021 Consensus Statements, the proportion of ATs feeling fairly confident or very confident in implementing the recommendations (scores of 4 or 5 on the 5-point ordinal scale).

The exposures of interest for both RQ3 and RQ4 included: 1) gender (man vs. woman); 2) years of practice as AT (grouped as <10, 10–19, 20–29, ≥30); 3) advanced degree (yes vs. no); 4) direct employment by HS or district (yes vs. no); 5) HS student enrollment (under 1000 vs 1000 and over); and 6) region, based on the Grundstein et al. [24] metrics of heat risk (Region I vs. II vs. III). Race/ethnicity was not examined due to low cell counts of those that did not identify as White/non-Hispanic. Also, age was not examined due to its high correlation with years of practice as AT (r = 0.83).

Prevalence ratios (PR) and 95% confidence intervals (CI) were calculated for these comparisons. For years of practice as AT, all groups were compared to the referent group of <10 years). All 95%CI excluding 1.00 were deemed statistically significant. Given the relatively small sample size potentially leading to underpowered analyses, alongside reporting statistically significant findings, we also reported those findings we deemed as near significant. We defined this as effect estimates with: 1) moderate associations (PR ≥1.40 or ≤0.71); [25] and 2) 95%CI that included 1.00, but 3) had a lower bound ≥0.90 or an upper bound ≤1.10.

## Results

### Sample characteristics

Among the sample of 116 HS ATs, the average years [± standard deviation (SD)] of age and AT practice were 46±11 and 23±11, respectively. Most ATs had an advanced degree (64.7%, n = 75), were directly employed by the HS or district (53.4%, n = 62) and identified as men (56.9%, n = 66) and White/non-Hispanic (88.8%, n = 103; Table 1). Most ATs worked at HSs that had at least 1000 students enrolled (60.3%, n = 70). In addition, participating ATs represented all three heat regions.

**Table 1:** Characteristics of participating high school athletic trainers.

Characteristic	n	%	Characteristic	n	%
Age in years^a^			Advanced degree^b^		
<30	9	7.8%	Yes	75	64.7%
30–39	20	17.2%	No	41	35.3%
40–49	39	33.6%	Employed by HS or district directly^c^		
50–59	39	33.6%	Yes	62	53.4%
≥60	9	7.8%	No	54	46.6%
Gender			HS student enrollment		
Man	66	56.9%	Under 1000	46	39.7%
Woman	50	43.1%	*<500*	*19*	*16*.*4%*
Identified as White / non-Hispanic^d^			*500–999*	*27*	*23*.*3%*
Yes	103	88.8%	1000 students and over	70	60.3%
No	13	12.3%	*1000–1999*	*49*	*42*.*2%*
Years of practice as AT			*≥2000*	*21*	*18*.*1%*
<10	13	11.2%	Heat region location^e^		
10–19	29	25.0%	I	30	25.9%
20–29	42	36.2%	II	43	37.1%
≥30	32	27.6%	III	43	37.1%

^a^ Correlation between age in years and years AT practice is 0.83.

^c^ Of the 54 not employed by HS or district directly: local medical system (i.e., hospital, PT clinic) n = 49; and independent contracting n = 6.

^d^ Of the 13 not identifying as White/non-Hispanic: Black/non-Hispanic n = 3; White/Hispanic n = 2; Asian/non-Hispanic n = 2; and Other n = 8.

^b^ Advanced degrees could originate from fields of athletic training, exercise science, kinesiology, sport administration, public health, health administration.

^e^ Heat safety region categorizations originate from Grundstein et al.; The mild Region 1 included the Pacific Coast and northern portions of the US; the moderate Region 2 included the midsection of the US; the hot Region 3 included the Southern US.

### Research question 1

**Fig 1:**
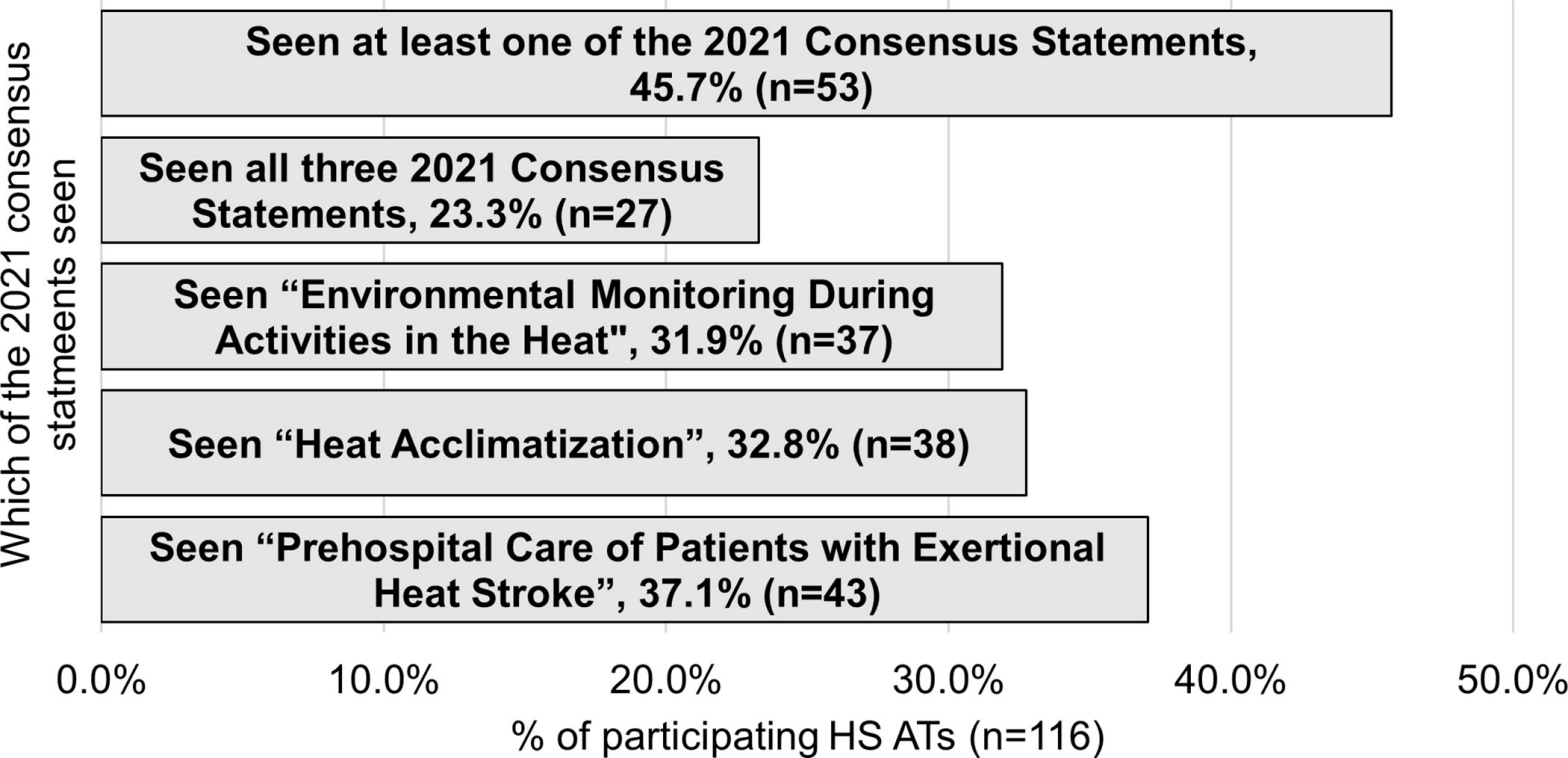
Percentages of participating HS ATs (n = 116) that had seen the 2021 Consensus Statements.

Overall, 53 ATs (45.7%) reported having seen at least one of the 2021 Consensus Statements (Fig 1). In addition, 27 ATs (23.3%) reviewed all three publications. The most reviewed publication was “Prehospital Care of Patients with Exertional Heat Stroke” (n = 43), followed by “Heat Acclimatization” (n = 38), and “Environmental Monitoring During Activities in the Heat” (n = 37). Of the 63 (54.3%) that had not seen the 2021 Consensus Statements, common codes identified for not seeing them included: not aware they were published (n = 22), have yet to read them (n = 19), and believed they could not access the journal (i.e., JAT) (n = 10; Table 2). For the last code, participants noted believing that they needed to be a paying member of NATA to access JAT materials and/or that JAT was behind a paywall as with non-open-access journals. In addition, three participating ATs explicitly noted they desired better delivery of the notice of the publications, such as direct emails with the PDFs or symposium-like events to better present them.

**Table 2:** Codes regarding why ATs (n = 63) had not seen the 2021 Consensus Statements.

Code	n	%	Example quote (Participant gender and age)
Unaware they were published	22	34.9%	“Didn’t know they existed” (Woman, 34) “Was unaware there were new recommendations” (Women, 26)
Have yet to read them	19	30.2%	“Got the email, never opened the link to read it” (Woman, 30) “Have not read it, not sure anything has changed from position statement” (Man, 54)
Believed they could not access journal	10	15.9%	“Was unable to access the journal via my email/websites” (Man, 59) “Not an NATA member to get the article” (Woman, 43)
Desired better delivery / notice of publication of recommendations	3	4.8%	“There are no emails that simply state what is available in that month’s journal. Normally the email has so much other junk in it, that I delete it” (Woman, 45)
Will read now that they realized they were published	3	4.8%	“I will use this link to review the updated recommendations” (Woman, 34)
The recommendations not as conversative as current guidelines	1	1.6%	“Heat guidelines in our state high school league tend to be more conservative than NATA position statement” (Woman, 37)
Missing data	14	22.2%	

### Research question 2

Of the 53 ATs reported having seen at least one of the 2021 Consensus Statements, 67.9% (n = 36) of ATs believed they would be very (n = 17) or fairly (n = 19) confident in implementing the recommendations at their HS (Table 3). Among those who felt very or fairly confident, common codes reported for feeling confident to implement the 2021 Consensus Statements pertained to their schools being proactive in ensuring EHI prevention and management were up to date (n = 18) and support from athletics constituents (n = 8). Supportive athletic constituents noted included coaches (n = 3), school administration (n = 2), and the school district (n = 2). Among those noting they were not at all, slightly, and somewhat confident (n = 17), common codes reported for feeling less confident in implementing the 2021 Consensus Statements pertained lack of support from athletics constituents (n = 9) and pushback to the use of rectal thermometers (n = 3). Non-supportive athletic constituents noted included school administration (n = 5), coaches (n = 2), and the school district (n = 2).

**Table 3:** Codes regarding why ATs who had seen at least one of the 2021 Consensus Statements (n = 53) felt confident/not confident in implementing them.

Codes	All (n = 53)		Level of confidence^a^				Example quote (Participant gender and age)
			Fairly and very confident (n = 36)		Not at all, slight, and somewhat confident (n = 17)		
	n	%	n	%	n	%	
School was proactive in ensuring up-to-date EHI prevention and management	18	34.0%	18	50.0%	0	0.0%	“We have coaches meetings every fall to go over emergency procedures and protocols so all coaches are aware of how to handle each situation if ATC is not available” (Woman, 56) “We go over EHI for training every year. We also have to teach it to our coaches to make sure they can follow guidelines and know what to do in an emergency” (Woman, 33)
Lack of support from athletics constituents	13	24.5%	4	11.1%	9	52.9%	“Lack of Administrative and School Physician Support. Their attitude is still in the 1980s” (Man, 64) “Resistance from School Legal Dept” (Man, 44)
Support from athletics constituents	8	15.1%	8	22.2%	0	0.0%	“Our school district allows the athletic trainers to make the medical decisions regarding EHI so that we can diagnose and treat properly” (Man, 54) “I have no push back from the school district, school admin or coaches” (Woman, 42)
Pushback to use of rectal thermometers	4	7.5%	1	2.8%	3	17.6%	“We are NOT approved for rectal temperatures in any circumstances by our school administration/ school board…Call 911 sooner is the information I received” (Woman, 52) “District refuse to allow invasive temp monitors” (Man, 53)
Unique circumstances	5	9.4%	3	8.3%	2	11.8%	“I work at 6300 feet above sea level, I have a loose heat policy, because it does not happen that often in my area…and during that time July, I am not contracted to be working” (Man, 48) “My schools unique practice locations are a potential barrier” (Man, 45)
Lack of finances	2	3.8%	1	2.8%	1	5.9%	“Finances to afford cooling tubs and have adequate recovery drinks or even being lucky to have cold water at some practices makes it more difficult” (Woman, 45)
Have yet to fully review 2021 Consensus Statements	1	1.9%	0	0.0%	1	5.9%	“I have not thoroughly reviewed the material / recommendations” (Man, 55)
Missing data	10	18.9%	6	16.7%	4	23.5%	

^a^ Perceived level of confidence in being able to implement 2021 Consensus Statements was based on 5-point-ordinal scale ranging from “not at all confident” to “very confident

### Research question 3

The groupings of ATs with the highest proportions that saw at least one of the 2021 Consensus Statements included those: with 10–19 years of practice as an AT (62.1%, n = 18/29); employed directly by a HS or district (53.2%, n = 33/62); and with an advanced degree (52.0%, n = 39/75); Table 4). Among those ATs with <10 years of practice, only one AT (7.7%) reported seeing at least one of the 2021 Consensus Statements; Table 5).

**Table 4:** Proportions of ATs (n = 116) that had seen at least one of the 2021 Consensus Statements.

Characteristic	n within category	Had seen at least one of the 2021 Consensus Statements		Prevalence Ratio (95%CI)
		n	%	
Gender				
Man	66	32	48.5%	1.
Woman	50	21	42.0%	0.87 (0.58, 1.31)
Years of practice as AT				
<10	13	1	7.7%	1.
10–19	29	18	62.1%	8.07 (1.20, 54.19)*
20–29	42	21	50.0%	6.50 (0.97, 43.77)
≥30	32	12	37.5%	4.88 (0.70, 33.77)
Advanced degree^a^				
No	41	14	34.1%	1.
Yes	75	39	52.0%	1.52 (0.94, 2.45)
Employed by HS or district directly^b^				
No	54	20	37.0%	1.
Yes	62	33	53.2%	1.44 (0.94, 2.18)
HS student enrollment				
Under 1000	46	19	41.3%	1.
1000 students and over	70	34	48.6%	1.18 (0.77, 1.79)
Heat region location^c^				
I	30	14	46.7%	1.
II	43	22	51.2%	1.10 (0.68, 1.77)
III	43	17	39.5%	0.85 (0.50, 1.44)

* statistically significant (i.e., 95%CI excludes 1.00)

^a^ Advanced degrees could originate from fields of athletic training, exercise science, kinesiology, sport administration, public health, health administration.

^b^ Of the 54 not employed by HS or district directly: local medical system (i.e., hospital, PT clinic) n = 49; and independent contracting n = 6

^c^ Heat safety region categorizations originate from Grundstein et al.; The mild Region 1 included the Pacific Coast and northern portions of the US; the moderate Region 2 included the midsection of the US; the hot Region 3 included the Southern US.

**Table 5:** Proportions of ATs (n = 116) that had seen all three of the 2021 Consensus Statements.

Characteristic	n within category	Had seen all three 2021 Consensus Statements		Prevalence Ratio (95%CI)
		n	%	
Gender				
Man	66	14	21.2%	1.
Woman	50	13	26.0%	1.23 (0.63, 2.37)
Years of practice as AT				
<10	13	0	0.0%	n/a ^a^
10–19	29	9	31.0%	
20–29	42	9	21.4%	
≥30	32	9	28.1%	
Advanced degree^b^				
No	41	7	17.1%	1.
Yes	75	20	26.7%	1.56 (0.72, 3.38)
Employed by HS or district directly^c^				
No	54	9	16.7%	1.
Yes	62	18	29.0%	1.74 (0.85, 3.55)
HS student enrollment				
Under 1000	46	11	23.9%	1.
1000 students and over	70	16	22.9%	0.96 (0.49, 1.87)
Heat region location^d^				
I	30	8	26.7%	1.
II	43	11	25.6%	0.96 (0.44, 2.10)
III	43	8	18.6%	0.70 (0.29, 1.65)

^a^ Analyses could not be conducted due to all in the referent group of <10 years having not seen all three 2021 Consensus Statements.

^b^ Advanced degrees could originate from fields of athletic training, exercise science, kinesiology, sport administration, public health, health administration.

^c^ Of the 54 not employed by HS or district directly: local medical system (i.e., hospital, PT clinic) n = 49; and independent contracting n = 6

^d^ Heat safety region categorizations originate from Grundstein et al.; The mild Region 1 included the Pacific Coast and northern portions of the US; the moderate Region 2 included the midsection of the US; the hot Region 3 included the Southern US.

When assessing differences in proportions between groups by AT characteristic, the only statistically significant difference was found within years of practice as an AT: the proportion of ATs having seen at least one of the 2021 Consensus Statements was higher among those with 10–19 years of practice compared to those with >10 years of practice (62.1% vs. 7.7%, PR = 8.07, 95%CI: 1.20–54.19; Table 4). Near-significant findings included: those having an advanced degree compared to those without (52.0% vs. 34.1%, PR = 1.52, 95%CI: 0.94–2.45); and those being directly employed by a HS or district versus those who were not (53.2% vs. 37.0%, PR = 1.44, 95%CI: 0.94–2.18). No statistically significant or near-significant findings were found when comparing proportions of participating HS ATs having seen all three 2021 Consensus Statements (Table 5).

### Research question 4

Of the 53 participating HS ATs that had seen at least one of the 2021 Consensus Statements, the groups with the highest proportions feeling fairly or very confident in implementing them included those: aged under 40 years (85.7%, n = 6/7); identifying as women (81.0%, n = 17/21); and working with a HS with student enrollment of 1000 and over (79.4%, n = 27/34; Table 6). When assessing differences in proportions between groups by AT characteristic, the only statistically significant difference was found by HS student enrollment: the proportion of ATs working in HSs with student enrollment of 1000 and over was higher compared to those working in HSs with under 1000 students (79.4% vs. 47.4%, PR = 1.68, 95%CI: 1.01–2.77). No near-significant findings were detected.

**Table 6:** Proportions of ATs reporting that they had seen at least one of the 2021 Consensus Statements (n = 53) that felt fairly or very confident in implementing them.

Characteristic	n within category	Felt fairly confident or very confident in implementing the recommendations		Prevalence Ratio (95%CI)
		n	%	
Gender				
Man	32	19	59.4%	1.
Woman	21	17	81.0%	1.36 (0.96, 1.94)
Years of practice as AT				
<10	1	1	100.0%	n/a ^a^
10–19	11	7	63.6%	
20–29	21	15	71.4%	
≥30	20	13	65.0%	
Advanced degree^a^				
No	14	10	71.4%	1.
Yes	39	26	66.7%	0.93 (0.63, 1.39)
Employed by HS or district directly^b^				
No	20	12	60.0%	1.
Yes	33	24	72.7%	1.21 (0.80, 1.83)
HS student enrollment				
Under 1000	19	9	47.4%	1.
1000 students and over	34	27	79.4%	1.68 (1.01, 2.77)
Heat region location^c^				
I	14	8	57.1%	1.
II	22	15	68.2%	1.19 (0.70, 2.04)
III	17	13	76.5%	1.34 (0.79, 2.26)

^a^ Analyses could not be conducted due to all in the referent group of <10 years having been fairly or very confident

^a^ Advanced degrees could originate from fields of athletic training, exercise science, kinesiology, sport administration, public health, health administration.

^b^ Of the 54 not employed by HS or district directly: local medical system (i.e., hospital, PT clinic) n = 49; and independent contracting n = 6

^c^ Heat safety region categorizations originate from Grundstein et al.; The mild Region 1 included the Pacific Coast and northern portions of the US; the moderate Region 2 included the midsection of the US; the hot Region 3 included the Southern US.

## Discussion

Multiple research- and evaluation-based frameworks [19–21] indicate a need for constant examination of the effectiveness and implementation of prevention strategies within health-related outcomes. This exploratory study gauged the level of exposure to the “Roundtable on Preseason Heat Safety in Secondary School Athletes” publications (i.e., the 2021 Consensus Statements) [16–18]. The study observed nearly half of participants reported having seen at least one of the 2021 Consensus Statements. Those who reported not having seen any of the Consensus Statements discussed being unaware of the publications. Variations were also found in terms of ATs’ viewing of the 2021 Consensus Statements and their level of confidence in implementing them.

It is important to note that the study had a low response rate (2.5%). This was lower than what was expected and we suspect this may have been due to the need to prioritize COVID-19-related sporting event modifications while dealing with potential work-related burnout and reductions in psychosocial health [26, 27]. Furthermore, this study was an add-on to a larger multi-method parent study focused on information seeking and implementation practices related to EHI among HS and collegiate Ats [22]. The intent was to gauge HS ATs’ initial familiarity with the recently introduced 2021 Consensus Statements in a timely manner. Thus, given the nature of the study and the subsequent sample size, caution must be taken with interpretations.

Nonetheless, the findings may highlight a myriad of factors affecting an AT’s ability to be exposed to prevention strategies. As posited in the socio-ecological framework [28, 29], it is important to consider the multiple levels of influence and the points of intervention to ensure proper dissemination and implementation of best practices such as that contained within the 2021 Consensus Statements. Further, follow-up studies are needed to verify the current study findings.

### Visibility of the 2021 consensus statements and associated characteristics (RQ1 and RQ3)

Approximately half of participants noted having seen at least one of the 2021 Consensus Statements (and a quarter had reviewed all three). Data collection for this study occurred within seven months of the 2021 Consensus Statements’ publications. Thus, it is important to contextualize this evaluation as one focused on the initial roll-out. In the time since data collection, continued and increased dissemination via formal (e.g., NATA-based educational pushes) and informal (e.g., word-of-mouth) processes may have increased visibility to date. However, our study highlights the need for improved strategies for early and immediate dissemination of best-practice documents.

Reasons noted for not having seen the 2021 Consensus Statements included those on the individual level (e.g., have not read them yet) but also those that highlighted the interaction of levels on the socio-ecological framework [28]. For example, some ATs noted the need for better delivery of notice of the publications; thus, improving organizational communication across membership can help to improve individual beliefs about access to resources. Future release efforts could include a variety of strategies, including short specific announcements tied in with online trainings or sessions presented at state/regional and national conferences.

Variations in the proportions of participating ATs having seen at least one of the 2021 Consensus Statements further highlight the influence of certain individual and organizational factors. There was potential evidence that visibility was greater among those with more years of practice, advanced degrees, and direct employment with their HS/school district. The disparity in knowledge acquisition based upon the tenure of AT experience may highlight the need to consider various dissemination processes. More “senior” ATs may assist in the dissemination of future Consensus Statements by connecting less experienced ATs with such guidelines.

It is also important to highlight that such discussions related to the implementation of prevention strategies is not exclusive to the 2021 Consensus Statements. Barriers across the levels of the socio-ecological framework have been identified as related to the adoption of Emergency Action Plans, including school location, policy mandates, and support from key constituents (e.g., athletic director, school leader) [30]. Further, evaluations of initial roll-out phases within national campaigns related to the COVID-19 vaccine and diabetes prevention have identified differences in adherence at individual and community levels [31–33]. Thus, the consideration of the multiple levels of influence posited by the socio-ecological framework must be integral with the initial roll-out and adoption of prevention practices led and assisted by ATs.

### Confidence in implementing the 2021 Consensus Statements and associated characteristics (RQ2 and RQ4)

Approximately two-thirds of participants who reported seeing the 2021 Consensus Statements felt fairly or very confident in implementing the associated recommendations at their HS. While the current study did not examine confidence by individual consensus statement or specific components within each statement (or whether actual implementation occurred), it is plausible that some components may be easier to implement than others. Previous research focused on pedagogical approaches have noted teachers felt more confident in implementing certain portions of curriculum [34, 35]. Considering this, future research should work to identify areas of focus.

Previous knowledge transfer-based research [36] has identified dissemination strategies to be: passive (e.g., mass communication of information without no tailoring of messages and/or targeted audiences); or active (e.g., targeted and inclusive of numerous resources such as continuing education and user feedback, and often perceived as a more effective dissemination strategy) [37]. Participants noted the mostly perceived passive dissemination strategies for learning about the 2021 Consensus Statements. As a result, increased efforts inclusive of active dissemination strategies are warranted. For example, previous research has noted differences in exertional heat stroke preparedness by region (based on various environmental factors across and within states) [38]. Thus, areas with lower levels of compliance, such as states located within relatively cooler areas [38] (e.g., West Coast, New England, and the Northern US [24]) may need dissemination efforts focused on the relevance of the 2021 Consensus Statements despite being located in areas that are perceived not to be at risk for EHI. In contrast, warmer areas may require more in-depth information related to circumstances that ensure best practices can be implemented in all scenarios.

At the same time, research has also noted that passive dissemination strategies for clinical recommendations may be effective, particularly when clinicians have a good baseline knowledge of the adverse outcome and are aided by opinion leaders who advocate for the application of the clinical recommendations [39]. As a result, evidence-based best practice documents such as the 2021 Consensus Statements may benefit from multiple levels of influence, as posited by the socio-ecological framework [28]. In the current study, factors associated with ATs’ confidence in implementation were at the interpersonal and organizational levels; this included support school district representatives, HS administrators, coaches, and parents. The findings reiterate previous research findings regarding constituent support [12, 40, 41] and highlight a multi-level approach is needed for the dissemination and implementation of best practices. Implementation of new guidelines is not solely carried out by the AT alone. All constituents need to have buy-in. From the organizational level, the NATA can help to inform all constituents of updated policy. State and national HS athletics associations should also provide support in the dissemination to constituents.

### Strengths and limitations

Our study’s sample was inclusive of ATs with varying sociodemographics, backgrounds, and employment types. However, the low sample size may have resulted in findings that are not generalizable to the total population of ATs practicing in HS settings. As previously noted, the study’s low response rate may be due to external factors related to workloads during the COVID-19 pandemic. Further, the distribution of demographics varies from that of recent BOC data, although this is inclusive of non-HS settings as well (e.g., universities/colleges, clinical settings, professional sports, performing arts) [42]. Also, findings may not be generalizable to non-HS settings nor HS settings lacking AT access [43].

Statistics generated from this study were done within seven months of the 2021 Consensus Statements’ release and may not represent current states of implementation. However, it is important to gauge the initial onset of dissemination to better understand how to improve efforts related to future consensus statements. Further, additional studies are needed to continue ascertaining both confidence in and actual implementation. Next, coding of open-ended responses was performed by two of the study team (ZYK, JCD). As with such analyses, biases may arise from coders’ previous experiences with topics examined. It was with this that the two coders were chosen to ensure varying levels of exposure to the study topic. Last, more in-depth interviews likely would provide additional information regarding the factors associated with having seen, feeling confident in implementing, and actual implementation of the 2021 Consensus Statements. Nonetheless, the findings may provide contexts that can aid the continued dissemination processes of these and future consensus statements. In closing, the study’s strengths and limitations highlight the need for evaluation to be a constant, continuous, and evolving process.

## Conclusion

This exploratory study observed that over half of surveyed HS ATs have not seen the 2021 Consensus Statements. The reasons noted for not seeing them highlight the need to continue improving messaging about access to updated best-practice recommendations, particularly emphasizing that JAT is open-access online. Further, one-third of those ATs who had seen the 2021 Consensus Statements did not feel confident in being able to implement them. Although a relatively small study, the findings underscore the need for continued efforts to identify and implement strategies that will aid proper implementation. Opportunities are also needed at the organizational level so that there is institutional knowledge. These includes assistance in creating evidence-based active and passive dissemination strategies.
